# Respiratory Epithelium Lined Cyst of the Maxilla: Differential Diagnosis

**DOI:** 10.1155/2017/6249649

**Published:** 2017-09-28

**Authors:** C. P. Martinelli-Kläy, S. Chatelain, F. Salvado, T. Lombardi

**Affiliations:** ^1^Laboratory of Oral & Maxillofacial Pathology, Oral and Maxillofacial Pathology Unit, Division of Oral Maxillofacial Surgery, Department of Surgery, University Hospitals of Geneva, University of Geneva, Geneva, Switzerland; ^2^Center for Interdisciplinary Research Egas Moniz (CiiEM), Health Sciences Institute, Monte da Caparica, Portugal

## Abstract

Maxillary cysts, including the cysts lined by respiratory epithelium, can present a diagnostic challenge. We report an unusual case of a maxillary cyst on an endodontically treated tooth #16, in which the cavity was totally lined by a respiratory epithelium. The patient, a 35-year-old male, presented with a generalized chronic periodontitis and complained of a pain in the tooth #16 region. A periodontal pocket extending to the root apices with pus coming out from the gingival was found. A combined endodontic periodontal was observed on a panoramic radiography. CBCT-scan revealed a well-circumscribed radiolucent lesion at the apices of the distobuccal root of the 16. A communication with the right maxillary sinus cavity and a maxillary and ethmoidal sinusitis were also observed. The lesion was removed and histological examination revealed a cyst lined exclusively by respiratory epithelium. Ciliated and rare mucous cells were also observed. The diagnosis could evoke a surgical ciliated cyst mimicking the radicular cyst but the patient has no previous history of trauma or surgery in the maxillofacial region. It could also be an unusual radicular cyst in which the stratified squamous epithelium was destroyed by inflammation and replaced by a respiratory epithelium of the maxillary sinus.

## 1. Introduction

The maxillary cysts lined by pseudostratified columnar epithelium (respiratory epithelium) can show different pathologic conditions and hinder the diagnosis, thus challenging the clinician and pathologist. These lesions include mucocele of the maxillary sinus and surgical ciliated cyst. The latter develops after a surgical procedure, such as maxillary sinus surgery (e.g., Caldwell-Luc), orthognathic surgery, and trauma caused by dental extraction [1–3]. In some cases, a radicular cyst can also be included in the differential diagnosis of these lesions. In fact, although its cavity is lined by a nonkeratinized stratified squamous epithelium, it can be partially or totally lined by a respiratory epithelium [4–7].

We report the case of an unusual cyst on the maxillary right first molar (tooth #16) region, in which the cavity was totally lined by respiratory epithelium. Interestingly, no previous history of surgical treatment of the sinus, trauma, or dental extraction was observed.

## 2. Case Presentation

**Figure 1:**
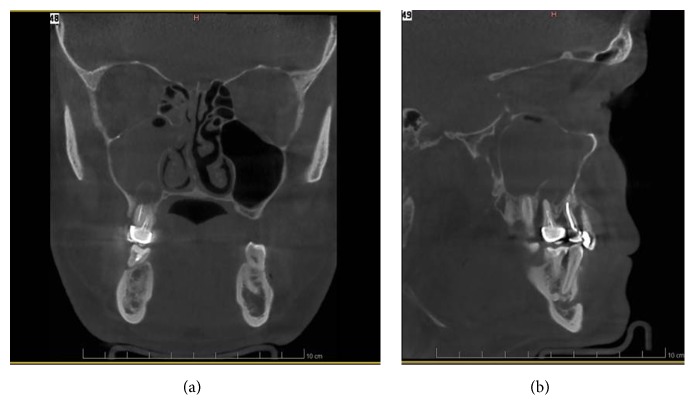
Coronal (a) and sagittal (b) CBCT-scan: well-defined hypodense lesion involving a distal and apical region (endo-perio lesion) of the endodontically treated right maxillary first molar. Maxillary and ethmoidal sinusitis and a perforation of the lesion into the right maxillary sinus cavity are also observed.

**Figure 2:**
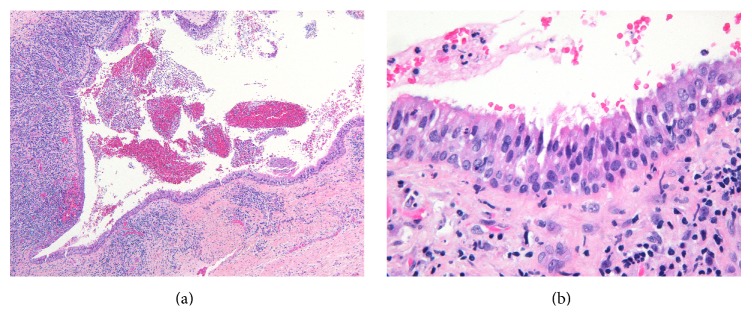
Histological aspect of radicular cyst: (a) cystic cavity containing hemorrhagic exudate and neutrophils lined exclusively by ciliated pseudostratified columnar epithelium. The wall of the cyst consists of fibrous connective tissue with an intense chronic inflammatory infiltrate. (HES, ×6.4). (b) High magnification showing the ciliated pseudostratified columnar epithelium and inflammatory infiltrate represented by lymphocytes, plasma cells, and some neutrophils (HES, ×60).

We report the case of a 35-year-old healthy male who consulted our Oral Surgery, Implantology and Pathology Emergency Department with a chief complaint of pain in the posterior maxillary right region. He reported no history of trauma or surgery in the maxillofacial region and was not known for recurrent sinusitis. The clinical examination revealed a generalized periodontitis. Tooth #16 presented a periodontal pocket extending to the root apices with pus coming out from the gingival sulcus. The mobility of the teeth was grade 3, the vitality was negative, and the percussion was positive. The patient was not swollen and did not have any systemic symptomatology. A severe generalized horizontal bone loss associated with local vertical lesions and furcation involvement in the first quadrant was seen on the panoramic radiography. The diagnosis of a combined endodontic periodontal lesion was inferred. The Cone-Beam Computed Tomography (CBCT) revealed a communication of 6 mm in the anteroposterior axis, of the apices of the root of tooth #16 with the right maxillary sinus cavity. A well-circumscribed lesion that protrudes in the maxillary sinus is described at the apices of the distobuccal root of tooth #16. The right maxillary and anterior ethmoidal sinus are opacified and the peripheral mucosa thickened. A deviation of the nasal septum on the right is also outlined (Figures 1(a) and 1(b)). Tooth #16 was extracted and the lesion which was attached to the root apices was removed entirely. The histological analysis showed a cystic cavity lined exclusively by respiratory epithelial tissue, containing scarce mucous and ciliated cells. The cyst wall consisted of fibrous connective tissue containing an intense chronic inflammatory infiltrate mainly represented by lymphocytes and plasma cells (Figures 2(a) and 2(b)). Some dystrophic calcifications were also observed (not shown). The cystic content was hemorrhagic and contained varied amount of inflammatory cells represented by neutrophils.

## 3. Discussion

In this particular case, the differential diagnosis of the maxillary cyst in which the cavity is fully lined by respiratory epithelium should include the surgical ciliated cyst or an unusual radicular cyst. In both cases, a resorption of the maxillary sinus floor can happen and cause the communication between the cyst and the maxillary sinus cavity.

The surgical ciliated cyst is a cystic lesion which develops after a surgical procedure such as maxillary sinus surgery (e.g., Caldwell-Luc), orthognathic surgery, and trauma caused by dental extraction. They are locally aggressive and may resemble a tumour. Bone expansion, pain, or discomfort in the maxillary region and fistulization in some cases can be present. Radiographically, they can be unilocular or multilocular radiolucent or radiopaque with a well-defined margin and surrounding sclerotic edge in the floor of the maxillary sinus, with or without bony perforation [1–3]. In some cases, the surgical ciliated cyst can involve the apices of the maxillary second premolar or first or second molar and mimic a radicular cyst [3]. According to literature, the development of this cyst would be caused by an entrapment of the sinus mucosa in the wound after the surgical procedure followed by an inflammatory process that would stimulate epithelial proliferation. Finally, the expansion of the cyst would be caused by the osmotic pressure difference [3].

Among the odontogenic cysts of the jaws, the radicular cyst (also called periapical cyst or apical periodontal cyst) is the most common. Radiographically, it shows a well-defined radiolucent area around the apex of a tooth root. Although it is usually asymptomatic, the cyst can cause pain after a secondary infection. The cyst arises from an inflammation at the root apices of a tooth secondary to pulp necrosis, which stimulates cell proliferation from the remains of odontogenic epithelial rests of Malassez. Caries and trauma can be responsible for radicular cyst formation [8] Histologically, the radicular cyst is represented by a cavity lined by a nonkeratinized stratified squamous epithelium showing variable hyperplasia, spongiosis, and exocytosis of neutrophils. Its fibrous wall can contain variable amounts of chronic inflammatory cells represented mainly by lymphocytes and plasma cells [8].

Nevertheless, the cystic cavity can in some cases be partially or totally lined by a respiratory epithelium characterized by ciliated pseudostratified columnar epithelium with mucous cells [4–7]. The exact origin of this epithelium still remains unclear. Three possibilities may be considered: (1) migration of cells from the maxillary sinuses or from the nasal cavity, (2) metaplasia of the stratified squamous epithelium, or (3) differentiation from totipotent cells present in the jawbones [9]. According to Ricucci et al., 2014 [7], 49 out of 167 apical periodontitis lesions were analyzed and diagnosed as cysts but only 4 of them were lined by a ciliated columnar epithelium. All of these 4 cases occurred in patients with untreated maxillary molars. One cyst only was completely lined by columnar ciliated epithelium. Mucous-producing cells were also observed. Among 205 radicular cysts studied by Takeda et al., 2005 [10], 37 presented mucous cells and 22 showed ciliated cells in epithelial lining. However, no radicular cysts completely lined by a respiratory epithelium were found. In another study, only 3 radicular cysts out of 256 apical periodontitis lesions were lined by ciliated columnar epithelium. Only one was entirely lined by ciliated columnar cells. The 3 cases concerned lesions in the maxillary premolar region [5].

We reported a cyst case on the maxillary right first molar (tooth #16), in which the cavity was totally lined by respiratory epithelium. This lesion could correspond to a special surgical ciliated cyst mimicking a radicular cyst [3] but the patient has no previous history of trauma or surgery in the maxillofacial region. It is not possible to rule out an unusual radicular cyst. The periapical inflammation could lead to sinusitis and a perforation of the maxillary sinus floor. If this happened, the stratified squamous epithelium that lined the radicular cyst would be destroyed and partially or totally replaced by a respiratory epithelium originating from the sinus [5, 7, 11].
